# Limited impact of intratumour heterogeneity on molecular risk assignment in endometrial cancer

**DOI:** 10.18632/oncotarget.16067

**Published:** 2017-03-10

**Authors:** Manouk van Esterik, Inge C. Van Gool, Cor D. de Kroon, Remi A. Nout, Carien L. Creutzberg, Vincent T.H.B.M. Smit, Tjalling Bosse, Ellen Stelloo

**Affiliations:** ^1^ Department of Pathology, Leiden University Medical Centre, Leiden, The Netherlands; ^2^ Department of Gynaecology, Leiden University Medical Centre, Leiden, The Netherlands; ^3^ Department of Radiation Oncology, Leiden University Medical Centre, Leiden, The Netherlands

**Keywords:** endometrial cancer, intratumour heterogeneity, molecular markers, prognostic, risk stratification

## Abstract

**Introduction:**

Individual prediction of tumour behaviour based on molecular markers may refine adjuvant treatment strategies in endometrial cancer (EC). As these molecular alterations are determined in a small tumour fraction, high intratumour heterogeneity may interfere with correct risk prediction. This study aimed to investigate to which extent intratumour heterogeneity exists for molecular markers and whether it affects the molecular risk assignment in EC.

**Methods:**

Forty-nine ECs (three tumour blocks/case) were selected with alterations in *POLE* (n=10), *CTNNB1* (n=8), p53 (n=10), mismatch repair (n=11), L1CAM (n=10), and ECs without any of these markers (n=9). Nine ECs carried more than one molecular marker. All 147 blocks were analysed for *POLE* exonuclease domain and *CTNNB1* exon 3 mutations, and for p53, mismatch repair and L1CAM protein expression. All blocks were assigned to a favourable, intermediate or unfavourable risk group, based on a molecular risk assignment.

**RESULTS:**

Concordance between the three tumour blocks for *POLE* and *CTNNB1* mutational status, and p53, mismatch repair and L1CAM protein expression was found in 100% (48/48), 95.9% (47/49), 93.9% (46/49), 98.0% (48/49), and 91.8% (45/49) of tumours, respectively. These discordances were found in a total of nine cases (18.4%). The intratumour heterogeneity impacted the risk assignment in five cases (10.2%).

**Conclusion:**

Intratumour heterogeneity of prognostic molecular markers in EC without morphologic heterogeneity is uncommon among three tumour fractions, affecting the molecular risk allocation in a limited number of cases. This low intratumour heterogeneity facilitates the implementation of the molecular risk assignment, advocating its use in clinical decision making.

## INTRODUCTION

Endometrial cancer (EC) is the fourth most common malignancy among women in the Western world [1, 2]. Surgery is the primary therapy for endometrial cancer followed by tailored adjuvant therapy [3]. Although recommendations for adjuvant treatment are currently based on clinical and histopathological prognostic factors, over- and undertreatment of EC patients remains. Therefore, recent studies focused on (epi) genetic characteristics to improve prediction of individual patients’ risk of recurrence.

The Cancer Genome Atlas (TCGA) identified, supported by other independent studies, promising prognostic molecular markers in EC: polymerase epsilon (*POLE*) exonuclease domain mutations, *CTNNB1* mutations, mismatch repair (MMR) deficiency, and *TP53* mutations [4–9]. Additionally, three large studies have demonstrated that L1 cell adhesion molecule (L1CAM; CD171) expression in more than 10% of tumour cells is a strong independent predictor for distant recurrences in EC [10–12]. As a result, based on an extensive integrated analysis of these molecular markers, two molecular integrated risk assignments, combining clinicopathological and molecular risk factors, have been proposed [6, 7]. In one of these assignments, similarly to the other, p53-mutant-like, L1CAM-positive, and substantial lymphovascular space invasion (LVSI) tumours were designated to have an unfavourable risk. MMR-deficient and *CTNNB1*-mutant were considered at intermediate risk, while *POLE*-mutant tumours and tumours with no specific molecular profile are designated favourable [5–7].

Molecular risk prediction in EC patients, once implemented, will be based on molecular analysis in a small portion of the tumour. In a cancer, however, multiple subclones can be present that differ in phenotype and/or genotype, resulting in a high degree of intratumour heterogeneity [13]. Such intratumour heterogeneity may interfere with correct risk prediction.

Studies on intratumour heterogeneity of the aforementioned molecular markers are sparse [13–22]. Some studies have reported low rates of subclonal p53 and MMR protein expression within one or multiple blocks of the same hysterectomy specimen [23–27]. Furthermore, in 92% of *TP53*-mutant cases, the mutation was present in the majority of tumour cells based on the cancer cell fraction [28]. In addition, previous studies have determined for a selected number of markers that molecular analysis on pre-operative endometrial cancer specimen are concordant with final hysterectomy specimen obtained at definitive surgical staging [10, 29–31]. In this study we aimed to investigate to which extent intratumour heterogeneity exists for the promising prognostic molecular markers and whether it affects the molecular risk assignment in EC.

## RESULTS

### Clinicopathological characteristics

In total, 49 ECs were included, of which the clinicopathological characteristics are shown in Table 1. The selected cases were representative of the endometrial cancer population with regard to age (median 65 years), FIGO stage (87.8% stage I+II) and tumour type (85.7% endometrioid), with a slight overrepresentation of grade 3 tumours (28.6%) [32]. The included ECs consisted of 10 *POLE*-mutant, 8 *CTNNB1*-mutant, 10 p53-mutant, 11 MMR deficient and 10 L1CAM-positive cases, and 9 cases without any of these markers. Nine ECs contained more than one molecular alteration.

**Table 1:** Clinicopathological patient characteristics (*N = 49*)

		N	%
Age			
	Median, range	65	46-78
FIGO stage 2009			
	I, II	43	87.8
	III, IV	6	12.2
Tumour type			
	Endometrioid	42	85.7
	(Pseudoglandular) Serous	7	14.3
Grade			
	1, 2	35	71.4
	3	14	28.6
Depth of myometrial invasion			
	< 50%	27	55.1
	≥ 50%	22	44.9
Lymphovascular space invasion			
	Substantial	7	14.3
	Absent/mild	42	85.7
Mutation status*			
	*POLE*-mutant	10	20.0
	*CTNNB1*-mutant	8	16.3
Altered protein expression*			
	MMR deficiency	11	22.4
	>10% L1CAM	10	20.0
	Mutant-like-p53	10	20.0
Molecular risk assignment*			
	favourable	17	34.7
	intermediate	19	38.8
	unfavourable	13	26.5

*Based on tumour block used for selection.

### Intratumour heterogeneity of the molecular markers

Concordance between the three tumour blocks for *POLE* and *CTNNB1*-mutational status, and p53, mismatch repair and L1CAM protein expression was found in 100% (48/48), 95.9% (47/49), 93.9% (46/49), 98.0% (48/49), and 91.8% (45/49) of tumours, respectively (Table 2). The ten discordances were identified in nine cases (9/49, 18.4%, Table 3). This intratumour heterogeneity was not reflected by differences in histological subtype, tumour grade or nuclear atypia within or between the tumour blocks.

**Table 2:** Intratumour concordance for the molecular markers

	Subgroup					
	POLE*	*CTNNB1*	p53	MMR	L1CAM	Total
Concordant	48 (100.0)	47 (95.9)	46 (93.9)	48 (98.0)	45 (91.8)	40 (81.6)
Discordant	0 (0.0)	2 (4.1)	3 (6.1)	1 (2.0)	4 (8.2)	9 (18.4)

*One case failed.

**Table 3:** Overview of the pathological characteristics and molecular markers in cases showing intratumour heterogeneity for at least one molecular marker

Case	Tumour block	FIGO stage	Tumour type	Grade	*POLE*	*CTNNB1*	MMR	L1CAM	p53	Sanger seq. *TP53*	Final assignment	Difference in assignment
1	a	II	EEC	1	wt	mut	intact	< 10%	wt	n.a.	intermediate	
	b		EEC	1	wt	wt	intact	< 10%	wt	n.a.	favourable	yes
	c		EEC	1	wt	wt	intact	< 10%	wt	n.a.	favourable	
2	a	IA	EEC	1	wt	mut	intact	< 10%	wt	n.a.	intermediate	
	b		EEC	1	wt	mut	intact	< 10%	wt	n.a.	intermediate	yes
	c		EEC	1	wt	wt	intact	< 10%	wt	n.a.	favourable	
3	a	IB	EEC	2	wt	wt	MLH1/PMS2 loss	< 10%	wt	n.a.	intermediate	
	b		EEC	2	wt	wt	MLH1/PMS2 loss*	< 10%	wt	n.a.	intermediate	yes
	c		EEC	2	wt	wt	intact	< 10%	wt	n.a.	favourable	
4	a	IIIA	Serous	3	wt	wt	intact	> 10%	mut	n.a.	unfavourable	
	b		Serous	3	wt	wt	intact	> 10%	mut	n.a.	unfavourable	no
	c		Serous	3	wt	wt	intact	< 10%	mut	n.a.	unfavourable	
5	a	IA	EEC	1	wt	wt	intact	> 10%	mut	n.a.	unfavourable	
	b		EEC	1	wt	wt	intact	> 10%	mut	n.a.	unfavourable	no
	c		EEC	1	wt	wt	intact	< 10%	mut	n.a.	unfavourable	
6	a	IB	EEC	1	mut	wt	intact	< 10%	wt	n.a.	favourable	
	b		EEC	1	mut	wt	intact	< 10%	wt	n.a.	favourable	no
	c		EEC	1	mut	wt	intact	> 10%	wt	n.a.	favourable	
7	a	IA	EEC	3	mut	wt	intact	< 10%	wt	mut	favourable	
	b		EEC	3	mut	wt	intact	< 10%	wt+	mut	favourable	uncertain
	c		EEC	3	n.a.	wt	intact	> 10%	mut	mut	uncertain	
8	a	IB	EEC	1	wt	mut	intact	< 10%	wt	mut	intermediate	
	b		EEC	1	wt	mut	intact	< 10%	wt+	mut	unfavourable	yes
	c		EEC	1	wt	mut	intact	< 10%	wt+	mut	unfavourable	
9	a	IB	EEC	3	wt	wt	MLH1/PMS2 loss	< 10%	wt	wt	intermediate	
	b		EEC	3	wt	wt	MLH1/PMS2 loss	< 10%	wt+	wt	intermediate	yes
	c		EEC	3	wt	wt	MLH1/PMS2 loss	< 10%	wt+	mut	unfavourable	

Abbreviations: seq, sequence; EEC, endometrioid; wt, wildtype; mut, mutation; n.a., not analysed,. *Subclonal loss.

wt+: wildtype with focal overexpression in <10% of the tumour.

Discordant *CTNNB1* mutation status was observed in two cases (cases 1 and 2), discordant MLH1/PMS2 protein expression in one case (case 3), discordant L1CAM expression in four cases (cases 4-7), and three cases showed discordant p53-mutant-like expression (cases 7-9) (Table 2, Table 3). Competitive allele-specific PCR for *POLE* exonuclease domain mutations revealed six discordant cases. Reanalysis of these discordant cases using Sanger sequencing revealed concordant findings among the three tumour blocks for *POLE* mutation status. However, Sanger sequencing could not be performed for one tumour block due to low DNA yield (case 7, block c).

**Figure 1:**
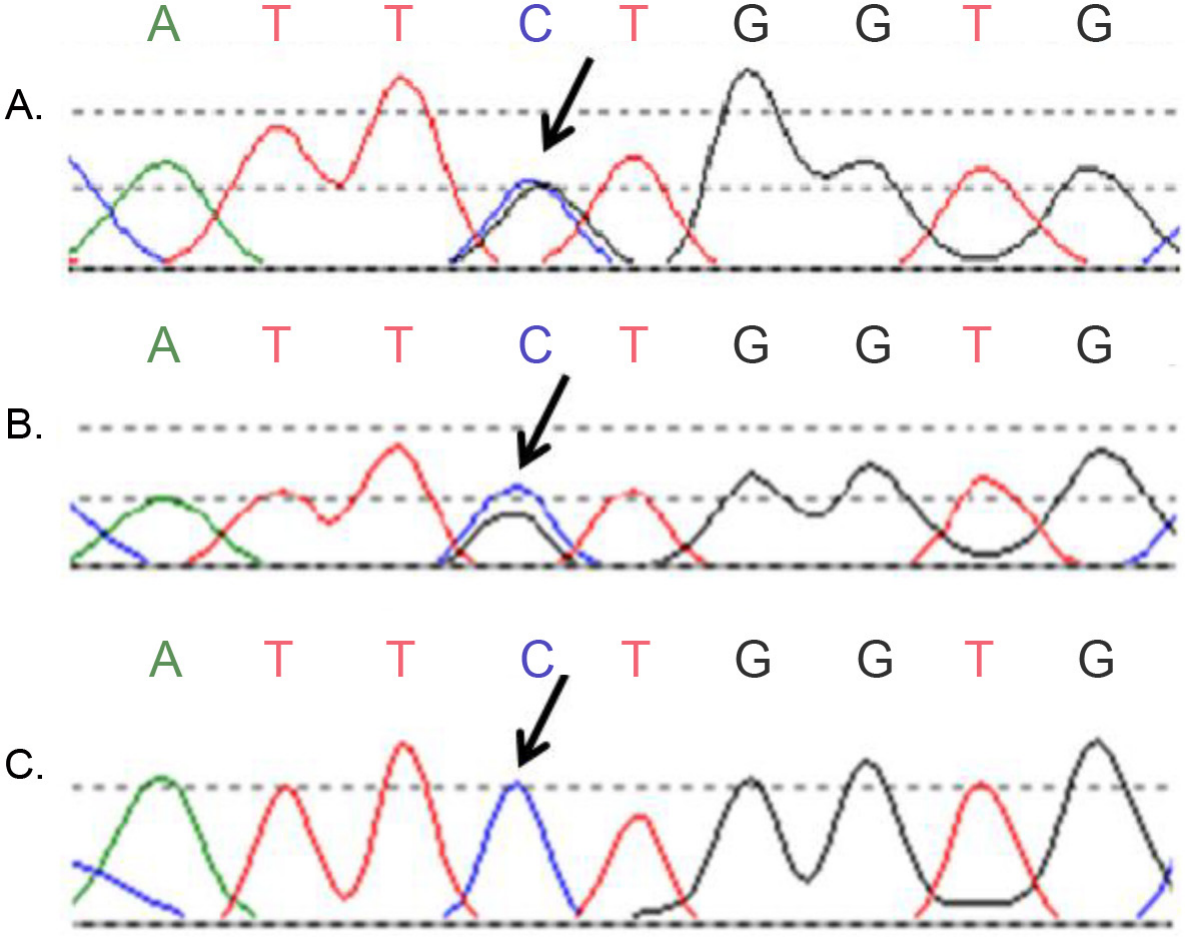
Discordant Sanger sequence results of CTNNB1 exon 3 (case 2) **(A, B)** Two tumour blocks showed a c.110C>CG mutation, indicated by the arrows; **(C)** The third tumour block of the same endometrial cancer patient was wildtype at the same position.

Sanger sequencing of *CTNNB1* exon 3 revealed discordant results in cases 1 and 2. In case 1, tumour block 1a harboured a c.94G>GT mutation with a variant allele frequency (25%), whereas no *CTNNB1* mutation was detected in block 1b and 1c. Case 1 was the only case from the initial group of nine cases without any of the markers that showed molecular alterations upon analysis of three tumour blocks. In case 2, a c.110C>CG mutation in *CTNNB1* was detected at high allelic frequency (80-100%) in tumour block 2a and 2b, but not in block 2c (Figure 1). To exclude contamination or exchange of DNA, we confirmed that all DNA originated from the same patient.

**Figure 2:**
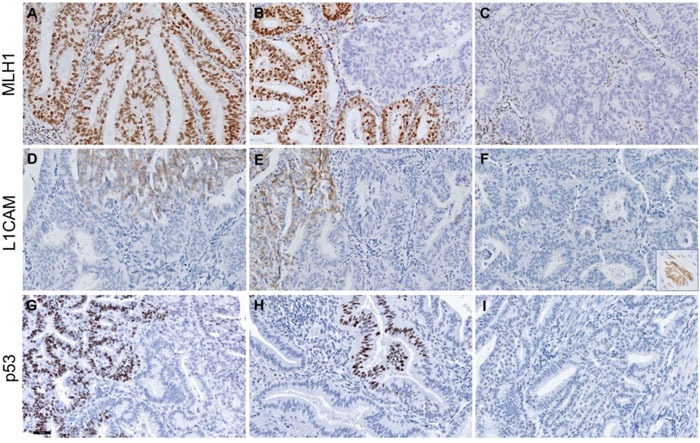
Representative figures of discordant MLH1, L1CAM and p53 protein expression in three endometrium carcinoma cases Upper panels show discordant MLH1 expression: intact MLH1 staining **(A)**, subclonal loss **(B)** and complete loss of MLH1 staining **(C)** in three blocks of case 3. Middle panels show discordant L1CAM staining in case 5: >10% L1CAM expression in two tumour blocks **(D, F)** and <10% in the third tumour block **(E)**. The inset in **(F)** represents a L1CAM-positive nerve (positive control) within the same slide. Lower panels show wildtype+ p53 staining patterns with focal overexpression in <10% of the tumour in two tumour blocks **(G, H)** and wildtype p53 expression in the third block **(I)** in case 8. Scalebar represents 50 μm.

Case 3 demonstrated discordant MMR protein expression (Figure 2A-2C). Tumour block 3c showed retained MLH1 and PMS2 expression, whereas tumour block 3a and 3b showed subclonal (15%) and complete loss of MLH1 and PMS2 expression, respectively. Moreover, three cases with complete loss of MLH1 and PMS2 expression in all three tumour blocks also displayed subclonal loss of MSH6 expression (10%, 20% and 90% of the tumour respectively) in one tumour block.

Cases 4-7 showed discordant L1CAM expression. In cases 4 and 5, more than 10% L1CAM expression was observed in two out of three tumour blocks (Figure 2D–2F). In cases 6 and 7, more than 10% L1CAM expression was observed in only one out of three tumour blocks.

Case 7 also showed discordant p53 expression: the first block demonstrated a p53-wildtype expression pattern, whereas the second block was scored ‘wildtype+’, because of focal p53 overexpression in a discrete geographical area of <10% of tumour cells. The third block showed a p53-mutant-like expression pattern in all tumour cells. Despite of the discordant p53 expression pattern found in the three blocks, the same pathogenic *TP53* mutation (c. 637C>CT) was identified in all three tumour blocks. For case 8, a p53-wildtype+ expression pattern was found in tumour blocks 8b and 8c (Figure 2G, 2H), whereas a p53-wildtype expression pattern was seen in block 8a (Figure 2I). Case 9 also showed p53-wildtype expression in one tumour block (9a), while blocks 9b and 9c demonstrated p53-wildtype+ expression. Sanger sequencing revealed a *TP53* pathogenic mutation (c.733G>GA) in all three blocks of case 8, while in case 9 only block 9c carried a pathogenic *TP53* mutation (c.524G>GA) [33, 34]. Due to a limited surface area, both p53-mutant and -wildtype tissue may have been microdissected of block 9b, resulting in a *TP53*-wildtype status.

### Molecular risk assignment

The intratumour heterogeneity found in the molecular markers affected the molecular risk assignment (Supplementary Figure 1) in five of 49 cases (10.2%, Table 3). Cases 1, 2 and 3 were assigned in the favourable or intermediate risk group depending on the tumour block used for molecular analysis. In these cases, risk assignment differed between the tumour blocks due to intratumour heterogeneity in *CTNNB1* mutation status and MMR protein expression. The discordant L1CAM expression within cases 4 and 5 did not affect the risk assignment, because of the homogeneous p53-mutant-like expression pattern in the tumour. The presence of a mutation in the *POLE* exonuclease domain determined cases 6 and 7 to be at favourable risk. Therefore, the discordancy in L1CAM and p53 protein expression did not affect the risk assignment. However, in cases 8 and 9, the risk assignment was influenced by focal p53-mutant-like overexpression and the presence of a pathogenic *TP53* mutation. These cases were assigned to the intermediate or unfavourable risk group depending on the tumour block used for molecular analysis.

## DISCUSSION

This is the first study which comprehensively examined the prevalence of intratumour heterogeneity of well-established prognostic molecular markers and its impact on molecular risk assignment in EC. This series of 49 cases showed, depending on the marker analysed, no or low intratumour heterogeneity among three tumour blocks for *POLE* and *CTNNB1* mutations, and p53, MMR and L1CAM expression (range of concordance rate: 92-100%). Similarly, previous studies showed that the molecular analysis on endometrial cancer preoperative specimens are concordant with final hysterectomy specimens obtained at definitive surgical staging [10, 29–31]. The intratumour heterogeneity in our study affected the integrated molecular risk assignment in only five cases. These findings suggest that testing one tumour tissue block from hysterectomy (or diagnostic) specimen is sufficient to provide a reliable molecular risk assessment in ECs.

Interestingly, we found discordant (subclonal) p53 mutant-like expression in three tumours. Although immunohistochemistry for p53 is routinely performed, only Feng et al. has described this p53 expression pattern [23]. As in the previous study, the majority of cases harboured a *TP53* mutation, predicted to be pathogenic, in the p53-mutant tumour cells. The study of Feng et al. showed that subclonal p53-mutant-like expression may be related to differences in differentiation in half of the cases, suggesting that these *TP53* mutations occurred at a later stage in tumour progression. No such histological differences were found in the present study within or between the tumour blocks of the same case showing subclonal p53-mutant-like expression. However, these three cases all showed an alteration in one of the other prognostic molecular markers, namely *POLE* mutation, *CTNNB1* mutation or MMR deficiency. This supports the possibility of *TP53* mutations as a late event, acting as a passenger alteration or aiding outgrowth of a more aggressive tumour. The concept is illustrated by case 7, a *POLE*-mutant tumour with subclonal p53-mutant-like expression due to a *TP53* mutations at p.(R213*). As this particular substitution corresponds to the mutational signature associated with *POLE* mutations, it may be a secondary event [35]. The role of subclonal p53-mutant-like expression in endometrial carcinogenesis and the corresponding clinical outcome of these patients remains to be elucidated. Pathologists should be aware of this expression pattern and depending on the biological behaviour, subclonal p53 mutant-like expression should be implemented in the scoring system.

In comparison to the other molecular markers, intratumour heterogeneity was found most frequently for L1CAM expression (∼8%). From a prognostic point of view, all but one of these cases with discordant L1CAM expression would have been classified as having an unfavourable risk regardless of L1CAM because of their p53-mutant-like expression or *TP53* mutation. Furthermore, L1CAM expression may be a predictive marker: patients with high L1CAM expression may potentially benefit from L1CAM antibody-mediated therapy [36]. Whether the intratumour heterogeneity of L1CAM impacts the efficacy of such targeted therapy remains to be investigated.

The level of intratumour heterogeneity, found in the present study, may be an overestimation due to our selection of relatively large tumours with at least three tumour blocks. In practice, many endometrial cancers are small, resulting in only one or two tumour blocks. Therefore, limited intratumour heterogeneity can be anticipated in these cases. Contrastingly, additional sampling of larger tumours would result in higher intratumour heterogeneity. Moreover, this study contained only ECs without morphological heterogeneity. Intratumour heterogeneity may be larger when selecting for cases with mixed morphology. However, studies have shown that the different histotype components in mixed tumours are commonly clonally related, sharing the same molecular markers [37–39]. Regardless of whether the reported level of intratumour heterogeneity is an over- or underestimation, it will be comparable to the levels documented for other implemented molecular markers, such as *HER2* in breast cancer (range 11-40%) [40–46].

In conclusion, intratumour heterogeneity of prognostic molecular markers in EC without morphologic heterogeneity is uncommon among three tumour fractions from hysterectomy specimen. The low levels of intratumour heterogeneity affected the molecular risk allocation in a limited number of cases. Therefore, prognostic molecular markers to guide adjuvant treatment decisions can be determined on a single representative sample of EC, facilitating their use in routine diagnostics.

## MATERIALS AND METHODS

### Case selection

ECs, previously molecularly profiled [7, 47], with a minimum of three available formalin-fixed paraffin-embedded (FFPE) tumour blocks were selected to give similar numbers of ±10 ECs with mutations in *POLE* and *CTNNB1*, alterations in MMR, p53 and L1CAM protein expression, and without any of these alterations. With this approach, our study set will be enriched for *POLE*-mutant ECs (20% of cases) in comparison to a random EC population (7-12% of cases) [48]. Only sporadic MMR-deficient tumours with proven *MLH1* promotor hypermethylation were selected. Haematoxylin-eosin (H&E) stained slides of each selected case were reviewed to randomly select three tumour blocks containing the highest percentage of tumour tissue. As a result of this random selection, in 27 (55%) of cases the tumour block used for initial molecular profiling was also included in this study [7, 47]. H&E slides of the selected tumour blocks were evaluated for histological features (i.e. tumour grade, histotype and nuclear atypia) by a gynaecopathologist.

In total, 43 ECs were selected from the pathology archive (2001-2015) of the Leiden University Medical Centre, the Netherlands and 6 ECs from the PORTEC-1 and -2 studies [49, 50] All women underwent a hysterectomy either with or without bilateral salpingo-oophorectomy followed by tailored adjuvant therapy according to the national clinical guidelines [51]. The clinicopathological data were obtained from the pathology reports and trial databases [49, 50]. This study was approved by the institutional review board.

### Immunohistochemical analysis

Immunohistochemistry on tissue slides (4 μM) was performed as previously described [10, 30, 52]. In brief, endogenous peroxidases were inactivated by 0.3% H_2_O_2_/methanol. Subsequently, antigen retrieval was achieved by microwave oven procedure in 10 mmol/l Tris-EDTA, pH 9.0. Sections were incubated overnight with primary monoclonal antibodies against L1CAM (clone 14.10, 1:500, Covance Inc.), p53 (clone DO-7; 1:1000; Neomarkers), MLH1 (clone ES05, 1:100; DAKO), MSH2 (clone FE11, 1:100, DAKO), MSH6 (clone EPR3945, 1:800, Genetex) or PMS2 (clone EP51, 1:25, DAKO). Sections stained for MLH1, MSH2 and PMS2 were incubated at room temperature with Envision FLEX+ Linker (DAKO) for 15 minutes. Thereafter, all sections were incubated and stained for 30 minutes using a secondary antibody (poly-HRP-GAM/R/R; DPV0110HRP; ImmunoLogic). Diamino-benzidine-tetrahydrochloride (DAKO) was used as a chromogen. Finally, the slides were counterstained with Mayer's haematoxylin, dehydrated and mounted.

For immunohistochemical analysis, slides were randomly numbered to ensure the two independent observers were blinded for the paired three tumour slides, patient characteristics, and the initial assigned status of the p53, L1CAM and MMR protein expression. Discrepancies were resolved by consensus under a multihead microscope. p53 was scored mutant-like if the whole tumour or a distinct geographical area of >10% of the tumour showed strong positive nuclear staining in >50% of tumour cells (diffuse or subclonal mutant-like) [53]. Cases were scored wildtype with focal overexpression if a discrete geographical area of <10% of the tumour showed strong positive nuclear staining (wildtype+). Sanger sequencing for *TP53* exon 5-8 mutations was performed as described previously for cases with ‘indefinite’ p53 expression (null staining), with subclonal mutant-like expression, with focal overexpression and ‘discordant’ cases in which the scores of the three tumour blocks differed [9]. The percentage of positive membranous L1CAM staining within the tumour was scored according to the scoring system for EC; 0%, <10%, 10-50%, or >50% [11]. Tumours were considered L1CAM positive if >10% L1CAM positivity was observed, based on prior studies [10, 11, 36]. Nerves from the deeper myometrium were used as internal positive controls and were identified in all cases. Tumours were considered MMR-deficient if tumour cells showed loss of nuclear staining of at least one of the MMR proteins either in the whole tumour or in a distinct geographical area (subclonal loss) with positive stromal cells, and MMR-proficient if tumour cells showed nuclear positivity for all mismatch repair proteins.

### DNA isolation and mutation analysis

To obtain tumour DNA, five sections (10 μM) were used to microdissect fragments of tumour with the aim to reach tumour percentage of >70%. Briefly, the sections were deparaffinised in xylene, rehydrated through a graded ethanol series, and stained with haematoxylin. The area of tumour tissue, marked on the H&E stained slide by a gynaecopathologist, was manually microdissected. After overnight proteinase K digestion, DNA isolation was performed according to manufacturer's instructions (NucleoSpin Tissue kit, Macherey-Nagel). Competitive allele-specific PCR (LGC Genomics) assays were used to screen for hotspot mutations in the *POLE* exonuclease domain [31]. Cases with discordant *POLE* mutation status among the three tumour blocks were reanalysed using Sanger sequencing to detect mutations in *POLE* exons 9 and 13 [31]. Sanger sequencing was also used to detect mutations in exon 3 of *CTNNB1* [8]. For the discordant *CTNNB1* cases, the Profiler Plus PCR Amplification kit (Applied Biosystems) was used to establish that the DNA isolated from three blocks have the same origin and to exclude contamination or exchange of DNA.

### Molecular risk assignment

The three tumour blocks of each case were assigned to a favourable, intermediate or unfavourable risk group, based on molecular markers included in a simplified integrated risk assignment (Supplementary Figure 1) [5–7]. P53-mutant-like and L1CAM-positive tumours were assigned as having an unfavourable risk. *CTNNB1*-mutant and MMR-deficient tumours were assigned as having an intermediate risk. *POLE*-mutant tumours and tumours with no specific molecular profile were assigned as having a favourable risk.

## SUPPLEMENTARY MATERIALS FIGURE



## Abbreviations

EC: endometrial cancer
FFPE: formalin-fixed paraffin-embedded
L1CAM: L1 cell adhesion molecule
MMR: mismatch repair
TCGA: The Cancer Genome Atlas
