# Supplementary material for: Limited impact of intratumour heterogeneity on molecular risk assignment in endometrial cancer
Source: Oncotarget. 2017 Mar 10;8(15):25542–51. doi: 10.18632/oncotarget.16067 (PMC5421949; doi:10.18632/oncotarget.16067)
Supplement: Supplementary file 1 [file oncotarget-08-25542-s001.pdf]

Limited impact of intratumour heterogeneity on molecular risk assignment in endometrial cancer

SUPPLEMENTARY MATERIALS

SUPPLEMENTARY FIGURE

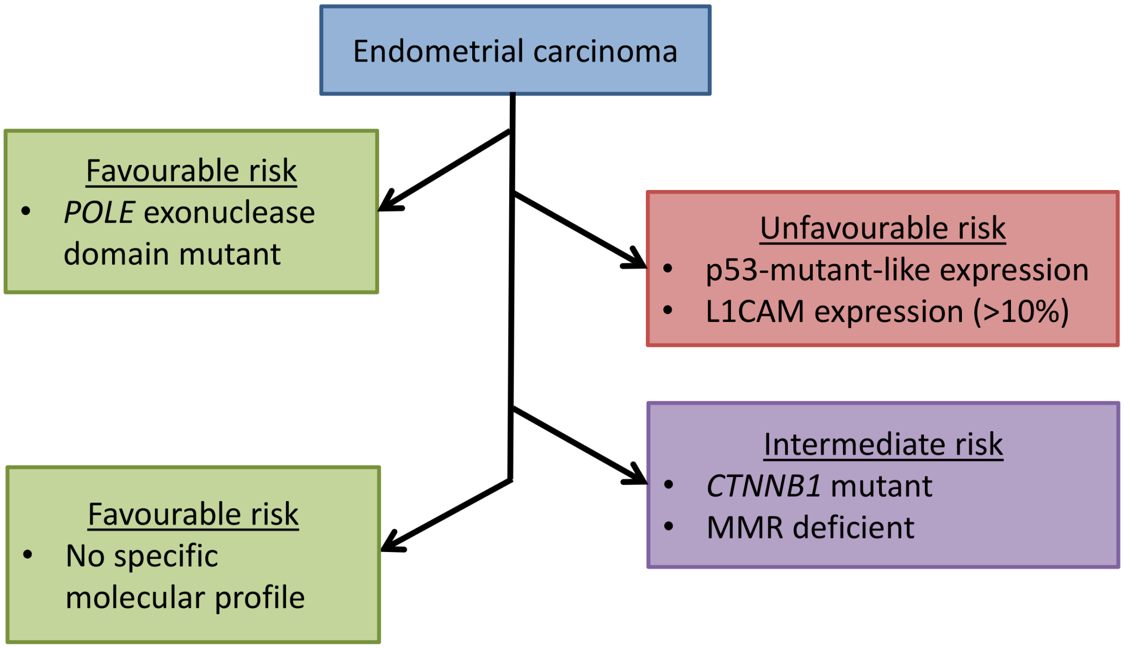

Supplementary Figure 1: The simplified integrated risk assignment in endometrial cancer.
